# Mining for Perchlorate Resistance Genes in Microorganisms From Sediments of a Hypersaline Pond in Atacama Desert, Chile

**DOI:** 10.3389/fmicb.2021.723874

**Published:** 2021-07-23

**Authors:** Jorge Díaz-Rullo, Gustavo Rodríguez-Valdecantos, Felipe Torres-Rojas, Luis Cid, Ignacio T. Vargas, Bernardo González, José Eduardo González-Pastor

**Affiliations:** ^1^Department of Molecular Evolution, Centro de Astrobiología (CSIC-INTA), Madrid, Spain; ^2^Polytechnic School, University of Alcalá, Alcalá de Henares, Spain; ^3^Faculty of Engineering and Sciences, Universidad Adolfo Ibáñez, Santiago, Chile; ^4^Center of Applied Ecology and Sustainability (CAPES), Faculty of Biological Sciences, Pontifical Catholic University of Chile, Santiago, Chile; ^5^Department of Hydraulic and Environmental Engineering, Pontificia Universidad Católica de Chile, Santiago, Chile; ^6^Centro de Desarrollo Urbano Sustentable (CEDEUS), Santiago, Chile

**Keywords:** perchlorate-resistance, oxidative stress, tRNA modification, DNA repair, protein damage, hypersaline environments, Atacama Desert, Mars

## Abstract

Perchlorate is an oxidative pollutant toxic to most of terrestrial life by promoting denaturation of macromolecules, oxidative stress, and DNA damage. However, several microorganisms, especially hyperhalophiles, are able to tolerate high levels of this compound. Furthermore, relatively high quantities of perchlorate salts were detected on the Martian surface, and due to its strong hygroscopicity and its ability to substantially decrease the freezing point of water, perchlorate is thought to increase the availability of liquid brine water in hyper-arid and cold environments, such as the Martian regolith. Therefore, perchlorate has been proposed as a compound worth studying to better understanding the habitability of the Martian surface. In the present work, to study the molecular mechanisms of perchlorate resistance, a functional metagenomic approach was used, and for that, a small-insert library was constructed with DNA isolated from microorganisms exposed to perchlorate in sediments of a hypersaline pond in the Atacama Desert, Chile (Salar de Maricunga), one of the regions with the highest levels of perchlorate on Earth. The metagenomic library was hosted in *Escherichia coli* DH10B strain and exposed to sodium perchlorate. This technique allowed the identification of nine perchlorate-resistant clones and their environmental DNA fragments were sequenced. A total of seventeen ORFs were predicted, individually cloned, and nine of them increased perchlorate resistance when expressed in *E. coli* DH10B cells. These genes encoded hypothetical conserved proteins of unknown functions and proteins similar to other not previously reported to be involved in perchlorate resistance that were related to different cellular processes such as RNA processing, tRNA modification, DNA protection and repair, metabolism, and protein degradation. Furthermore, these genes also conferred resistance to UV-radiation, 4-nitroquinoline-N-oxide (4-NQO) and/or hydrogen peroxide (H_2_O_2_), other stress conditions that induce oxidative stress, and damage in proteins and nucleic acids. Therefore, the novel genes identified will help us to better understand the molecular strategies of microorganisms to survive in the presence of perchlorate and may be used in Mars exploration for creating perchlorate-resistance strains interesting for developing Bioregenerative Life Support Systems (BLSS) based on *in situ* resource utilization (ISRU).

## Introduction

Perchlorate (ClO_4_^–^) is a soluble anion that consists of a chlorine atom surrounded by four oxygen atoms in a tetrahedral array. Perchlorate is a powerful oxidant, although under most environmental conditions, it is a highly stable anion (Urbansky, 1998). It is very soluble in water and perchlorate salts generally dissociate completely in aqueous solution. Thus, perchlorate adsorption to soil is very low and its mobility is mainly influenced by the hydrology of the environment (Coates and Achenbach, 2004). In addition, perchlorate anion can lead to very low eutectic temperatures when dissolved in water, ranging from −74 to −34°C, depending on the counter-cations (Marion et al., 2010). In Victoria Valley, one of the Antarctic Dry Valleys, where temperatures vary from −30 to −15°C, subsurface perchlorate-containing brines were discovered that supported a slow but active bacterial ecosystem (Kenig et al., 2016). Furthermore, perchlorate salts are very hygroscopic and can absorb moisture to form a liquid solution (Zorzano et al., 2009). As a result, perchlorate is thought to increase the availability of liquid water, at least locally and temporarily, in hyper-arid and cold environments.

Perchlorate is widespread in the global environment. It is a ubiquitous pollutant of water, soil and food and toxic for most known microorganisms and for humans (Kumarathilaka et al., 2016). It is a water contaminant resulting from anthropogenic perchlorate-containing products used in aerospace, industrial and military applications, such as fertilizers, explosives, fireworks or rocket fuels (Motzer, 2001; Backus et al., 2005) and the legal historical discharge of unregulated manufacturing waste streams (Coates and Achenbach, 2004). Furthermore, natural sources of perchlorate can be found mainly in arid environments, such as the Atacama Desert (Chile), where it is present in high concentrations associated with nitrate deposits (Vega et al., 2018). Perchlorate can be naturally formed by a photochemical process consisting of several reactions involving ozone oxidation, UV radiation, and electrical discharges (Catling et al., 2010). After formation in the atmosphere, perchlorate may reach the ground by precipitation, but given its high solubility and low adsorption to soil, it is rapidly transported to water sources. However, dry deposition could be also an important contributor to perchlorate accumulation, especially in arid desert environments (Andraski et al., 2014), such as the Atacama Desert or the Antarctic Dry Valleys, where the very infrequent precipitations may prevent perchlorate leaching and allow its accumulation in soils (Ericksen, 1981; Kounaves et al., 2010).

Large amounts of perchlorate (0.4–0.6% wt%) have been detected on the Martian surface regolith of Gale Crater and the Phoenix landing site (Hecht et al., 2009; Leshin et al., 2013; Kounaves et al., 2014). Perchlorate is thought to be the most abundant anion in Martian soil, and several processes have been proposed to explain these data (Schuttlefield et al., 2011; Martínez-Pabello et al., 2019). Moreover, the Mars Reconnaissance Orbiter has reported the presence of perchlorate salts at the seasonal flows on the warm Martian slopes, also called recurring slope lineae (RSL) (Ojha et al., 2015). It was suggested that RSLs occur during the warmest months on Mars due to the transient flow of liquid brine water at approximately −50°C (Chevrier and Rivera-Valentin, 2012; Oren, 2014). The presence of perchlorate salts in RSL, due to their ability of leading very low eutectic temperatures and their high hygroscopic capacity, supports the hypothesis that seasonal warm slopes are forming brine liquid water on contemporary Mars, which might suppose a refuge for halophilic prokaryotes (Davila et al., 2013; Oren et al., 2014). Nevertheless, the role for liquid brines in RSL formation is under discussion (Edwards and Piqueux, 2016; Dundas et al., 2017).

The toxicity of perchlorate is mainly due to its chaotropic activity. Perchlorate is one of the strongest chaotropic agents according to the Hofmeister scale (Zhang and Cremer, 2010). These molecules, when dissolved in water, can disrupt the hydrogen bonding network between water and macromolecules. This phenomenon entropically weakens the intramolecular hydrophobic interactions and promotes the denaturation of macromolecules. Therefore, perchlorate as a chaotropic agent may induce protein denaturation, destabilization of lipid bilayers, and precipitation of nucleic acids (Ingram, 1981; Hallsworth et al., 2003; Zhang and Cremer, 2010). Furthermore, due to its chaotropic activity and oxidative properties, perchlorate induces oxidative stress (Urbansky, 1998; Hallsworth et al., 2003) and was shown to cause DNA damage at low concentrations in testicle rat cells (Yu et al., 2019). In humans, perchlorate inhibits iodide uptake by the thyroid gland by blocking the sodium–iodide symporter. This inhibition reduces thyroid hormone production and the long-term reduction of these hormones can lead to hypothyroidism (Coates and Achenbach, 2004; Vega et al., 2018). This syndrome is especially dangerous for pregnant women, newborns, infants, and children, where thyroid function is key to normal growth, especially to neurocognitive development (Brent, 2014).

Due to its physicochemical properties, its widespread distribution in both terrestrial and Martian extreme environments and its toxicity, perchlorate has a major astrobiological and ecological interest at present. It is known that some microorganisms can reduce perchlorate for obtaining energy under anaerobic conditions. These microorganisms are called dissimilatory perchlorate-reducing bacteria (DPRB) and belong to the *Proteobacteria* phylum, *Dechloromonas* sp. and *Azospira* sp. being the best studied species (Thrash et al., 2007; Clark et al., 2012; Torres-Rojas et al., 2020). All DPRBs contain a perchlorate reductase (PcrAB) in the periplasm. This complex can reduce perchlorate and chlorate ions (ClO_4_^–^ or ClO_3_^–^) into chlorite (ClO_2_^–^), which is then dismutate into Cl^–^ and O_2_ by a chlorite dismutase (Cld) (Coates and Achenbach, 2004; Wang and Coates, 2017). However, non-canonical forms of perchlorate reduction have been proposed (Youngblut et al., 2016). Symbiotic perchlorate reduction has been reported, where one organism reduces perchlorate and a second one removes the ClO_2_^–^ (Clark et al., 2016). Furthermore, periplasmic nitrate reductases (pNar) and probably other dimethylsulfoxide reductases are supposed to be capable of reducing perchlorate, in a process called cryptic-perchlorate reduction. The microorganisms that may use this process must tolerate ClO_2_^–^ or transform it by unknown mechanisms. Most of them belong to the archaeal phyla Euryarchaeota and Crenarchaeota, for instance, the hyperthermophilic *Aeropyrum pernix* and the extreme halophilic *Haloferax mediterranei* (Youngblut et al., 2016). Those processes described above, and other unknown molecular mechanisms might be responsible for the high tolerance to perchlorate (0.5–1 M) exhibited by most of hyperhalophilic archaea (Oren et al., 2014).

On the other hand, several studies have reported the identification of novel perchlorate tolerant microorganisms which do not reduce perchlorate (Al Soudi et al., 2017; Beblo-Vranesevic et al., 2017; Matsubara et al., 2017; Heinz et al., 2020). Therefore, there must be additional mechanisms or biological adaptations that allow non-reducing perchlorate microorganisms to resist perchlorate. It is considered that resistance mechanisms to chaotropic agents may be related to protein stabilization, lipid metabolism or membrane composition (Hallsworth et al., 2003). However, information about perchlorate-resistance mechanisms is still largely missing in the literature. Only a recent research describes genes that confer resistance to UV and to perchlorate in *Escherichia coli*, possibly involved in DNA damage repair or protection, and that were isolated from a screening of metagenomic libraries of microorganisms from hypersaline environments to search for UV resistance genes (Lamprecht-Grandío et al., 2020). The study of novel perchlorate tolerant microorganisms may reveal information about those mechanisms, but only 0.1 to 1% of microorganisms are culturable on standard laboratory media (Rappé and Giovannoni, 2003). Culture-independent techniques may unveil information on novel resistance mechanisms of uncultured organisms, therefore, a functional metagenomic approach was used to identify novel genes involved in perchlorate resistance in microorganisms naturally exposed to this compound from sediments of a hypersaline pond in the Salar de Maricunga, at Atacama Desert (Chile) (Vega et al., 2018).

## Materials and Methods

### Bacteria Strains, Media and Culture Conditions

*E. coli* DH10B strain (Invitrogen, Waltham, MA, United States) was routinely grown in Luria-Bertani (LB) medium (Condalab, Madrid, Spain) at 37°C. The growth medium for transformed *E. coli* DH10B strains was supplemented with 100 μg mL^–1^ ampicillin (LB-Ap) to maintain the pBlueScript II SK (+) plasmid (pSKII+). When required, working concentrations of 5-bromo-4-chloro-3-indolyl-β-galactopyranoside (X-Gal) and isopropyl-β-D-1-thiogalactopyranoside (IPTG) were 40 μg mL^–1^ and 100 μM, respectively. For solid cultures, the growth medium was supplemented with agar (15 g L^–1^). Liquid cultures were shaken on an orbital platform operating at 200 r.p.m. For resistance assays, growth medium was supplemented with the minimal inhibitory concentration (MIC) of the different agents: 125 mM sodium perchlorate (NaClO_4_) (Thermo Fisher Scientific, Waltham, MA, United States), 0.2 μM 4-nitroquinoline-N-oxide (4-NQO) (Sigma-Aldrich) or 1 mM hydrogen peroxide (H_2_O_2_) (Sigma-Aldrich, St. Louis, MO, United States). MICs were established as the lowest sublethal concentration of the different toxic compounds for *E. coli* DH10B carrying empty pSKII+ vector (DH10B/pSKII+).

### Isolation of Metagenomic DNA From Sediments

Samples used in this study were collected from 1 m depth sediments from a hypersaline pond of the Salar de Maricunga (26°55′0.12′′ S 69°04′59.88′′ W), located in the Atacama Desert (Chile). This lake is 3,750 meters above sea level and its salinity is higher than 20% (w/v). The environment of the Salar de Maricunga is characterized by exceptionally arid conditions, having an annual precipitation of about 50 mm, and by being one of the highest nitrate deposits known in northern Chile (Vega et al., 2018). Nitrate deposits contains naturally occurring perchlorate anion, and in this region, it is accumulated and retained up to 70 g kg^−1^ due to an almost rainless climate with little or no rainwater that may drag perchlorate (Schilt, 1979; Ericksen, 1981).

Samples were maintained with RNAlater (Thermo Fisher Scientific, Waltham, MA, United States) and stored at −80°C until DNA isolation. After removing RNAlater by decantation, 1.8 μg of high-quality metagenomic DNA per g of sample was isolated using FastDNA Spin Kit for Soil and the FastPrep instrument (MP biomedicals, Irvine, CA, United States) following the manufacturer’s recommendations.

### Construction of Metagenomic Library

The construction of the metagenomic library was performed as previously reported (Mirete et al., 2007; González-Pastor and Mirete, 2010), using the high-copy number vector pSKII+ and the *E. coli* DH10B strain as host. Briefly, the metagenomic DNA was partially digested using the restriction enzyme *Sau*3AI. Completely *Bam*HI-digested pSKII+ vector was dephosphorylated using calf-intestinal alkaline phosphatase (Invitrogen) and phenolized. DNA fragments ranged from 2 to 8 kb and pSKII+ vector were finally purified directly from a 1% low-melting agarose gel using the QIAquick Gel Extraction kit (QIAGEN, Hilden, Germany). The purified pSKII+ and metagenomic DNA were ligated in a 1:6 molar ratio using T4 DNA ligase (Roche, Basel, Switzerland), incubating overnight at 16°C and then 20 min at 65°C to inactivate the enzyme. Dialyzed ligation was used to transform *E. coli* DH10B competent cells (Invitrogen) by electroporation using MicroPulser (Bio-Rad, Hercules, CA, United States) according to the manufacturer’s instructions. For library amplification, transformed cells were grown in liquid LB-Ap medium at 22°C until the culture reached exponential phase (approximately 20 h). The amplified library was supplemented with 20% glycerol (v/v) and stored at −80°C. Before and after amplification, transformed cells were selected on LB-Ap agar plates containing X-Gal and IPTG to estimate the amplification rate and the percentage of cells with recombinant plasmids (blue/white screening). To determine the average insert size of the library, plasmids from 72 random clones were isolated and digested using *Xba*I and *Xho*I restriction enzymes (Roche, Basel, Switzerland).

### Screening the Library for Perchlorate-Resistant Clones

The perchlorate-resistance phenotype was selected by plating 10^5^ recombinant cells from the amplified library on LB-Ap agar supplemented with 125 mM sodium perchlorate, a lethal concentration for DH10B/pSKII+. Plates were incubated for 48 h at 37°C, surviving colonies were considered putative perchlorate-resistant clones and were pooled together. Plasmid DNA was isolated from the pool and used to transform *E. coli* DH10B cells. This double transformation was performed to avoid spontaneous chromosomal mutations. New transformed clones were selected on LB-Ap agar plates and the phenotype of 200 randomly selected colonies was confirmed by patching each colony twice in LB-Ap agar supplemented with 125 mM sodium perchlorate. The perchlorate resistance of the clones with the best phenotypes was individually tested by a drop assay.

### Resistance Tests by Drop Assay

*Escherichia coli* DH10B recombinant clones with perchlorate-resistance phenotype were grown overnight individually in liquid LB medium at 37°C. The optical density of each culture (OD_600_) was adjusted to 1.0 and serial dilutions were performed to extinction. 10 μL drops of each dilution were sequentially inoculated on LB-Ap agar plates with sodium perchlorate, 4-NQO or H_2_O_2_ at the MICs previously established, or exposed to UV-B radiation (312 nm) at 2 W m^–2^ for 20 s using the irradiation chamber BS-02 UV/VIS (Opsytec Dr. Gröbel, Ettlingen, Germany). Drops were also inoculated on LB-Ap agar to verify that the cell viability of all cultures was similar. Plates were incubated at 37°C overnight for UV-B and H_2_O_2_ resistance tests, or for 48 h for perchlorate and 4-NQO tests. Images were taken using the precision DP70 CCD camera (Olympus). Survival percentages of each clone were calculated as the number of CFU mL^–1^ remaining after the different treatments divided by the initial CFU mL^–1^ DH10B/pSKII+ was used as a negative control. A negative result was registered when little or no growth of colonies was observed.

### *In silico* Analysis of Perchlorate- Resistant Clones

The environmental DNA fragments cloned into the plasmids of the perchlorate-resistant clones were sequenced on both strands with universal primers M13F and M13R and others for primer walking. For this purpose, the ABI PRISM dye terminator cycle-sequencing ready-reaction kit and an ABI PRISM 377 sequencer (Perkin-Elmer, Waltham, MA, United States) were used, according to the manufacturer’s instructions. Sequences were assembled and analyzed with EditSeq version 15.0 and SeqMan NGen version 12.0 (DNASTAR, Madison, WI, United States). Prediction of putative open reading frames (ORFs) was performed using ORF Finder available at the NCBI website^1^. For translation of the predicted ORFs, bacterial and archaeal codes were selected, allowing the presence of alternative start codons. Predicted ORFs longer than 75 bp were translated and used as queries in BLASTP (NCBI, Bethesda, MD, United States). The putative function was annotated based on their similarities to protein family domains by using Pfam, available at the European Bioinformatics Institute (EMBL-EBI)^2^, Simple Modular Architecture Research Tool (SMART)^3^ or Conserved Domains Database (CDD), available at the NCBI website^4^. Those sequences with an E-value higher than 0.001 in the BLASTP searches and longer than 300 bp were considered unknown. Transmembrane helices were predicted with TMpred^5^.

### Cloning of Candidate ORFs Involved in Perchlorate Resistance

The ORFs conferring perchlorate resistance were identified by subcloning each individual ORF from the original metagenomic clones in pSKII+ using specific primers (Supplementary Table 1). Subcloning was accomplished by PCR amplification of the ORFs using the following reaction mixture: 50 ng of plasmid DNA, 0.2 μM of each forward and reverse primers, 0.25 mM of an equimolar mix of the four deoxynucleoside triphosphates and 2.5 U of Pfu Turbo DNA polymerase (Stratagene), up to a total volume of 50 μL. The PCR amplification program used was as follows: 1 cycle of 5 min at 94°C; 30 cycles of 45 s at 94°C, 45 s at 54°C, and 10 min at 72°C; and finally, 1 cycle of 10 min at 72°C. A 150-bp region upstream of the start codon was also amplified to include their native expression sequences (promoters and ribosome binding sites). PCR amplification products were purified from a 1% low-melting agarose gel using the QIAquick Gel Extraction kit (QIAGEN, Hilden, Germany). PCRs products and pSKII+ plasmid were digested with the appropriate restriction enzymes (Roche). Digested PCR products were gel purified as explained above and ligated into pSKII+ keeping the same orientation as that of the original clone. In the case of truncated ORFs, subcloning was performed maintaining exactly the same join between the ORF and the pSKII+. *E. coli* DH10B cells were transformed with the ligation products and the resulting recombinant clones were tested for perchlorate-resistance by drop assay as described above.

### Cloning of Other Genes in *E. coli* DH10B

The *queF* genes from *E. coli* DH10B and *Bacillus subtilis* PY79, and the *yrbC* gene from *E. coli* DH10B were cloned and overexpressed in *E. coli* DH10B. DNA fragments containing their coding region and a 150 bp sequence upstream were amplified by PCR as previously described using specific primers (Supplementary Table 1). Amplified fragments were ligated to pSKII+ and the ligation product was used to transform *E. coli* DH10B cells. Recombinant clones were selected on LB-Ap agar plates supplemented with X-Gal and IPTG, and confirmed by restriction analysis and sequencing.

### Statistical Analysis

Statistical parameters, including value of n, mean and standard deviation (S.D.), are reported in Figures and Figure legends. One-way ANOVA followed by Dunnet *post hoc* analysis was used for statistical analysis using GraphPad Prism version 7.00 (GraphPad Software, La Jolla, CA, United States).

## Results

### Screening for Genes Involved in Perchlorate Resistance

To identify genes involved in perchlorate resistance, a functional metagenomics approach was carried out. For this, we constructed a library with metagenomic DNA isolated from sediments of a hypersaline pond of the Salar de Maricunga, in the Atacama Desert (Chile), one of the areas on Earth most naturally enriched in perchlorate. Perchlorate has been found in drinking water in Atacama Region at concentrations as high as 1480 μg L^−1^, whereas standards for drinking water range from 2 to 18 μg L^−1^ (Vega et al., 2018). However, perchlorate concentration is presumably increased in hypersaline lakes and brines, as in Salar de Bellavista, where perchlorate was found at 0.21 g L^−1^ (Ericksen, 1981). The metagenomic DNA was partially digested and cloned in the high-copy vector pSKII+ and the resulting plasmid library was hosted in the *E. coli* DH10B strain. Approximately 300,000 recombinant clones were obtained, with an average insert size of 1.5 kb, ranging from 0.5 to 4 kb, based on the restriction analysis of plasmids isolated from 72 random recombinant clones. About 450 Mb of environmental DNA was cloned in this library.

**FIGURE 1:**
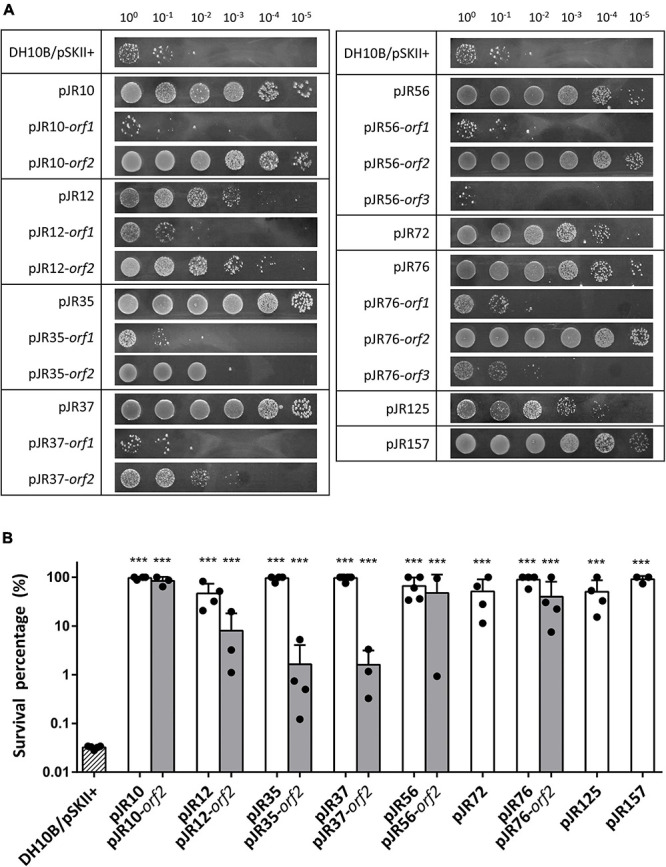
Drop assays **(A)** and survival percentage **(B)** of the nine complete clones and individual predicted ORFs involved in perchlorate-resistance. Cultures were grown overnight at 37°C, drops of 10-fold serial dilutions were plated on LB-Ap containing 125 mM of sodium perchlorate and plates were incubated for 48 h at 37°C. Survival percentage was calculated as the number of CFU mL^–1^ remaining after the treatment divided by the initial CFU mL^–1^. *E. coli* DH10B bearing empty pSKII+ (pBlueScript II SK +) vector was used as a negative control. One-way ANOVA followed by Dunnet *post hoc* analysis revealed the displayed significant differences against the negative control (***p < 0.001). Data represent the mean ± S.D (*n* ≥ 3).

Ten aliquots of 10^5^ cells of the amplified library were plated on LB-Ap agar containing 125 mM sodium perchlorate, a lethal concentration for the control strain DH10B/pSKII+. A total of about 1,000 perchlorate-resistant colonies were detected after incubation at 37°C overnight. To exclude clones with chromosomal mutations that could confer perchlorate resistance, all resistant colonies were pooled, plasmids DNA were extracted and used to transform DH10B cells. Recombinant colonies were selected on LB-Ap agar plates containing X-Gal and IPTG and the phenotype of 200 colonies was verified by patching onto LB-Ap agar containing 125 mM sodium perchlorate. The plasmids from 17 confirmed resistant clones were isolated and restriction analysis with *Xba*I and *Xho*I revealed a total of nine clones with unique restriction pattern. These plasmids clearly conferred perchlorate resistance on the resulting recombinant *E. coli* DH10B strains, which exhibited different resistance profiles in the drop assay test (Figure 1A). The quantification of perchlorate-resistance level showed significant differences in the survival percentage among the recombinant clones and the negative control DH10B/pSKII+. In the case of recombinant clones, survival percentage ranged from 47 to 100%, which corresponds to a 1400 to 3000-fold increase (Figure 1B). An additional drop assay was performed with 125 mM sodium chloride, a non-oxidative salt, to exclude that toxic effects of perchlorate were due to the increase in salinity by the addition of sodium perchlorate to the medium (Supplementary Figure 1).

### Identification of Genes Conferring Perchlorate Resistance

**FIGURE 2:**
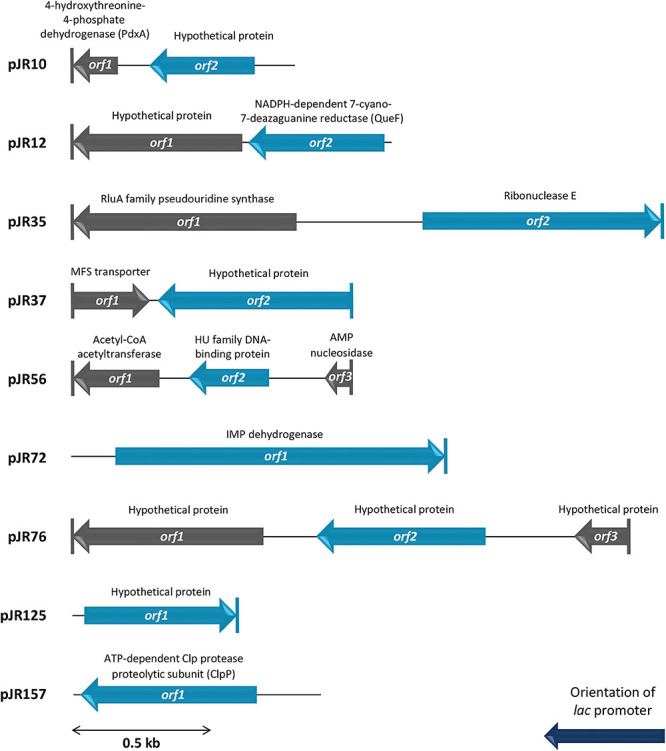
Schematic organization of the predicted ORFs identified in plasmids pJR10 to pJR157. Arrows indicate the locations and the transcriptional orientation of the ORFs in the different plasmids. Those predicted ORFs involved in perchlorate resistance are denoted by blue arrows. Truncated ORFs are indicated by a vertical bar at the corresponding end.

The complete double-stranded DNA sequences of each insert of perchlorate-resistant plasmids pJR10, pJR12, pJR35, pJR37, pJR56, pJR72, pJR76, pJR125, and pJR157 were obtained by primer walking. The gene organization of the inserts and the similarities, putative domains and protein family functions of the predicted encoded proteins are summarized in Figure 2 and Table 1. The length of the inserts ranged from 0.6 to 2.1 kb. The G + C content varied from 51 to 75%, indicating a diverse phylogenetic affiliation. Most of inserts showed a G + C content higher than 60%, which could be explained by their halophilic origin (Jones and Baxter, 2016; González-Torres and Gabaldón, 2018). The inserts of the nine plasmids harbored a total of 17 predicted ORFs, twelve of them truncated. Ten predicted ORFs encoded proteins similar to known proteins and seven encoded proteins similar to conserved hypothetical proteins. All predicted ORFs products were similar to proteins of different species of bacteria, mostly halophiles such as *Halofilum ochraceum* and *Coraliomargarita* sp. WN38, isolated from a marine solar saltern of Weihai (China) (Xia et al., 2017; Zhou et al., 2019), and *Roseibaca ekhonensis*, isolated from the hypersaline, heliothermal Ekho Lake (Antarctica) (Labrenz et al., 2009).

**TABLE 1:** Description of the perchlorate-resistant plasmids and their observed sequence similarities.

**ID clone (size [bp]) %_(G+C)_**	**GenBank accession no.**	**ORF^a^**	**Truncated end**	**Length (aa)**	**Closest similar protein (organism)**	**Accession no.**	**Length (aa)**	**Amino acid identity to the closest similar protein (%)**	**Putative domains**	**Putative function**
pJR10 (778) 67.1	MW924778	1	C-term	58	4-hydroxythreonine-4-phosphate dehydrogenase PdxA (*Acidobacteria bacterium*)	HBL28335.1	353	37/54 (69)	Pyridoxal phosphate biosynthetic protein PdxA, COG1995	PdxA catalyzes the fourth step in the pyridoxal 5′-phosphate (B6 vitamin) biosynthesis I pathway.
		**2**	**−**	**130**	**Hypothetical protein (*Acidobacteria bacterium*)**	**HBL28336.1**	**116**	**77/104 (74)**	**Sm-like superfamily, cl00259**	**Unknown.**
pJR12 (1136) 70.0	MW924779	1	C-term	237	Hypothetical protein (*Halofilum ochraceum*)	WP_070988095.1	361	173/237 (73)	Sortilin-Vps10 family, pfam15902	Unknown.
		**2**	**−**	**129**	**NADPH-dependent 7-cyano-7-deazaguanine reductase QueF (*Halofilum ochraceum*)**	**WP_070988098.1**	**129**	**101/129 (78)**	**NADPH-dependent 7-cyano-7-deazaguanine reductase QueF, pfam14489**	**Queuosine (Q) biosynthesis. Q is a modified nucleoside present at G34 of Asp, Asn, His and Tyr tRNAs**.
pJR35 (2133) 68.9	MW924780	1	C-term	268	RluA family pseudouridine synthase (*Halofilum ochraceum*)	WP_067562153.1	312	178/243 (73)	RluA family, COG0564	Modification of uridine to pseudouridine (Ψ) at certain positions of 23S RNA and tRNA.
		**2**	**C-term**	**504**	**Ribonuclease E (*Halofilum ochraceum*)**	**WP_067562152.1**	**1090**	**191/231 (83)**	**Ribonuclease E/G family, pfam10150**	**RNA decay and processing.**
pJR37 (1005) 75.2	MW924781	1	N-term	87	MFS transporter (*Marinobacter* sp. X15-166B)	WP_070056854.1	625	49/85 (58)	Lysophospholipid acyltransferases superfamily, cl17185	Acyltransferases of *de novo* and remodeling pathways of glycerophospholipid biosynthesis.
		**2**	**N-term**	**231**	**Hypothetical protein (*Sandaracinus amylolyticus*)**	**WP_053237473.1**	**260**	**113/236 (48)**	**Alanine dehydrogenase/PNT, C-terminal domain, smart01002**	**Unknown.**
pJR56 (999) 60.2	MW924782	1	C-term	104	Acetyl-CoA acetyltransferase (*Roseibaca ekhonensis*)	WP_121095031.1	389	94/104 (90)	Acetyl-CoA acetyltransferase family, COG0183	Lipid and steroid metabolism.
		**2**	**−**	**127**	**HU family DNA-binding protein (*Roseibaca ekhonensis*)**	**WP_121095029.1**	**96**	**88/96 (92)**	**Bacterial DNA-binding protein family, pfam00216**	**HU contributes to chromosomal compaction and maintenance of negative supercoiling.**
		3	N-term	29	AMP nucleosidase (*Roseibaca calidilacus*)	WP_072246791.1	490	28/28 (100)	AMP nucleosidase superfamily, cl35675	Reversible hydrolysis of AMP into D-ribose 5-phosphate and adenine.
pJR72 (1360) 62.6	MW924783	**1**	**C-term**	**395**	**IMP dehydrogenase (*Verrucomicrobia bacterium*)**	**TAN37780.1**	**497**	**260/383 (68)**	**IMP dehydrogenase family, pfam00478**	**Oxidation of IMP to XMP. Rate-limiting step in the *de novo* synthesis of guanine nucleotides.**
pJR76 (2092) 61.7	MW924784	1	C-term	237	Hypothetical protein DRQ48_04840 (*Gammaproteobacteria bacterium*)	RKZ71028.1	549	123/256 (52)	MMPL superfamily, cl21543	Unknown.
		**2**	**−**	**278**	**Hypothetical protein A2W69_03275 (*Gammaproteobacteria* bacterium RIFCSPLOWO2_02_47_7)**	**OGT65256.1**	**224**	**98/181 (54)**	**MlaC protein family, pfam05494**	**Unknown.**
		3	N-term	72	Hypothetical protein (*Methylophaga lonarensis*)	WP_009726618.1	139	40/69 (58)	Putative transmembrane protein (PGPGW), pfam09656	Unknown.
pJR125 (623) 69.2	MW924785	**1**	**C-term**	**193**	**Hypothetical protein (*Deltaproteobacteria bacterium*)**	**HCH63108.1**	**327**	**78/215 (36)**	**Hypothetical**	**Unknown.**
pJR157 (917) 51.8	MW924786	**1**	**−**	**212**	**ATP-dependent Clp protease proteolytic subunit (*Coraliomargarita* sp. *WN38*)**	**WP_110132180.1**	**192**	**162/192 (84)**	**ATP-dependent Clp protease family, COG0740**	**Involved in degradation of misfolded or dysfunctional proteins.**

*^*a*^ORFs involved in perchlorate resistance (analyzed by drop assay) are shown in boldface type.*

To determine which predicted ORFs were responsible for the perchlorate-resistance phenotype of each clone, they were individually subcloned into the pSKII+, expressed in the *E. coli* DH10B strain and tested for their resistance to sodium perchlorate by drop assay. The DH10B/pSKII+ strain and each original clone (harboring the complete environmental DNA fragment) were used as negative and positive control, respectively. The results showed that nine of the 17 predicted ORFs may be implicated in perchlorate-resistance (Figures 1A, 2), which induced in *E. coli* DH10B survival percentages ranged from 1.6 to 100%, which corresponds to a 50 to 3000-fold increase (Figure 1B). pJR10-*orf2*, pJR12-*orf2*, pJR35-*orf2*, pJR37-*orf2*, pJR72-*orf1*, pJR76-*orf2* and pJR125-*orf1* conferred perchlorate tolerance up to 150 mM sodium perchlorate, whereas the maximum tolerance induced by pJR56-*orf2* and pJR157-*orf1* was 175 mM sodium perchlorate (data not shown).

Plasmids pJR72, pJR125, and pJR157 contained a single ORF which might be responsible for the increased perchlorate resistance of the host. pJR72-*orf1* encoded for a protein truncated at C-terminal similar (68% identity) to an IMP dehydrogenase (IMPDH) from *Verrucomicrobia bacterium*, and pJR157-*orf1* encoded for a protein highly similar (84% identity) to the Clp protease proteolytic subunit (ClpP) from *Coraliomargarita* sp. WN38. None of them have never been associated to perchlorate resistance, but ClpP could enhance the degradation of misfolded, dysfunctional proteins produced by chaotropic effects of perchlorate. On the other hand, pJR125-*orf1* encoded for a C-terminal truncated protein slightly similar (36% identity) to a conserved hypothetical protein from *Deltaproteobacteria bacterium* without any functional domain or putative transmembrane domain.

In the case of plasmids pJR10, pJR12, pJR35, and pJR37, inserts harbored two predicted ORFs and only *orf2* could be responsible for the perchlorate resistance. pJR10-*orf2* encoded for a protein highly similar (74% identity) to a conserved Hfq-domain containing hypothetical protein from *Acidobacteria bacterium*, pJR12-*orf2* for a protein highly similar (78% identity) to a NADPH-dependent 7-cyano-7-deazaguanine reductase (QueF) from *H. ochraceum*, pJR35-*orf2* for a C-terminal truncated protein highly similar (83% identity) to a ribonuclease E (RNase E) also from *H. ochraceum*, and pJR37-*orf2* for a N-terminal truncated protein slightly similar (48% identity) to another conserved alanine dehydrogenase-domain containing hypothetical protein from *Sandaracinus amylolyticus*. These proteins, except the hypothetical protein encoded by pJR37-*orf2*, are related to RNA metabolism, which has never been previously associated to perchlorate resistance.

**FIGURE 3:**
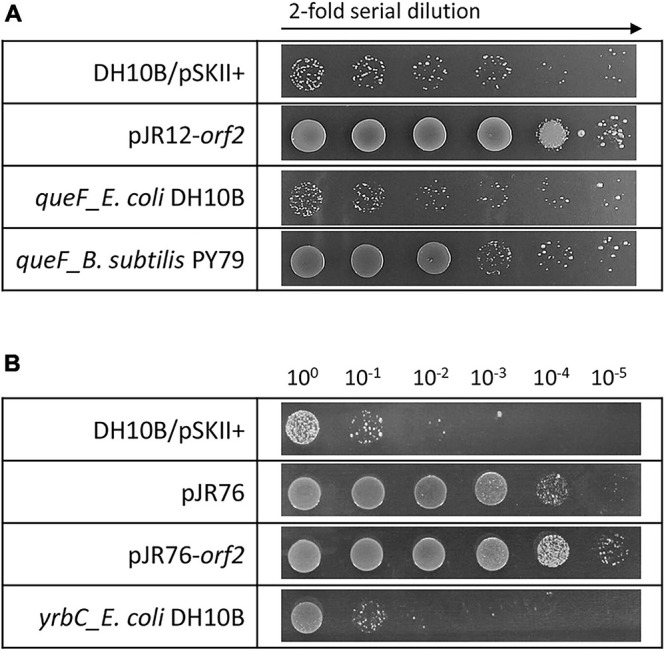
Drop assays in the presence of perchlorate of clones overexpressing: **(A)** pJR12-*orf2*, *E. coli* DH10B *queF* and *B. subtilis* PY79 *queF*, and **(B)** pJR76, pJR76-*orf2* and *E. coli* DH10B *yrbC*. Cultures were grown overnight at 37°C, drops of 2-fold **(A)** or 10-fold **(B)** serial dilutions were plated on LB-Ap containing 125 mM of sodium perchlorate and plates were incubated for 48 h at 37°C. *E. coli* DH10B bearing empty pSKII+ (pBlueScript II SK +) vector was used as a negative control.

The hypothetical protein encoded by pJR10-*orf2* contained a Hfq domain (Pfam PF17209) that belongs to the Sm-like superfamily (Pfam CL0527; CDD cl00259). This superfamily is composed of conserved domains of the core of RNA-binding proteins involved in a variety of RNA processing events including mRNA degradation and rRNA processing. In the case of Hfq domain, it is present in the RNA chaperone Hfq, an RNA-binding pleiotropic post-translational regulator of bacteria which mainly helps pair regulatory non-coding RNAs with complementary mRNA target regions (Dos Santos et al., 2019). In addition, QueF, encoded by pJR12-*orf2*, is involved in queuosine biosynthesis, a modified nucleoside present at the wobble anticodon position of certain tRNAs (Harada and Nishimura, 1972). QueF proteins are classified in two subfamilies: type I proteins, represented by *B. subtilis* QueF, and type II proteins, represented by *E. coli* QueF (Van Lanen et al., 2005). pJR12-*orf2* encoded for a protein similar to QueF enzyme of *H. ochraceum*, a Gram-positive microorganism. This protein was similar to the QueF of *B. subtilis* PY79 (52% identity), whereas showed no similarity to *E. coli* DH10B QueF, suggesting that it may belong to the type I QueF proteins. Overexpression tests of the *queF* genes representing these two subfamilies showed that perchlorate resistance was only conferred to *E. coli* DH10B strain when the *B. subtilis* PY79 *queF* gene was overexpressed but not with that of *E. coli* DH10B (Figure 3A). In addition, RNase E encoded by pJR35-*orf2* plays an important role in RNA processing and decay (Carpousis et al., 2009), whereas the hypothetical protein encoded by pJR37-*orf2*, that contained an alanine dehydrogenase/PNT, C-terminal domain (smart01002) is involved in the reversible reductive amination of pyruvate into alanine. It is important to note that pJR35-*orf2* and pJR37-*orf2* conferred lower perchlorate-resistance when cloned in *E. coli* DH10B compared to plasmids pJR35 and pJR37 that harbor the complete environmental DNA fragments (Figure 1A), which could suggest that proteins encoded by *orf1* are required for an optimal performance of the protein encoded by *orf2*.

In the case of plasmids pJR56 and pJR76, environmental inserts contained three predicted ORFs, and only *orf2* may be involved in the perchlorate resistance (Figures 1, 2). pJR56-*orf2* encoded for a protein highly similar (92% identity) to a DNA-binding protein HU from *Roseibaca ekhonensis*, and pJR76-*orf2* for protein similar (54% identity) to a conserved hypothetical protein from *Gammaproteobacteria bacterium* RIFCSPLOWO2_02_47_7. HU DNA-binding proteins are bacterial histone-like proteins that contributes to protect, compact and repair the DNA (Rouvière-Yaniv et al., 1979; Boubrik and Rouvière-Yaniv, 1995). Although they have never been associated to perchlorate resistance, HU proteins may protect against DNA damage produced by the chaotropic and oxidative effects of perchlorate. The hypothetical protein encoded by pJR76-*orf2* contained a MlaC domain (Pfam PF05494). This domain is present at MlaC protein, which is a component of the Mla pathway, an ABC transporter system that functions to maintain the lipid asymmetry of the outer membrane of Gram-negative bacteria. However, overexpression in the DH10B strain of the *E. coli* gene *yrbC*, which encodes for MlaC and constitutes an ortholog of the hypothetical protein encoded by pJR76-*orf2* (similarity of 46%), did not increase perchlorate resistance (Figure 3B). Thus, this hypothetical protein would play a different role.

In summary, we have retrieved from the microbial population of the Salar de Maricunga a total of nine novel putative genes that confer perchlorate resistance to *E. coli*. These genes encoded for proteins not previously reported to be involved in perchlorate resistance. Five of them encoded for proteins with known functions (pJR12-*orf2*, pJR35-*orf2*, pJR56-*orf2*, pJR72-*orf1*, and pJR157-*orf1*), but the other four genes (pJR10-*orf2*, pJR37-*orf2*, pJR76-*orf2*, and pJR125-*orf1*) encoded for conserved hypothetical proteins of unknown function.

### Perchlorate Resistance Genes Also Confer Resistance to Other Stress Conditions

As described before, perchlorate promotes proteins and nucleic acids denaturation, disorder of lipid bilayers, oxidative stress and DNA damage (Ingram, 1981; Hallsworth et al., 2003; Zhang and Cremer, 2010; Yu et al., 2019). To analyze whether the recombinant perchlorate-resistant clones and the predicted ORFs also conferred resistance to other stress conditions that produce those damages, the effects of exposure to UV irradiation, 4-NQO, and H_2_O_2_ were tested on them (Table 2).

**TABLE 2:** Cross-resistances showed by perchlorate-resistant clones and individual predicted ORFs.

**Plasmid**	**NaClO_4_**	**UV-B**	**4-NQO**	**H_2_O_2_**
pJR10	+ + + +	+ +	+ +	−
pJR10-*orf2*	+ + + +	+ + +	+ +	−
pJR12	+ + + +	+ +	−	+ +
pJR12-*orf2*	+ + +	+ ++	−	+ +
pJR35	+ + + +	+	++	−
pJR35-*orf2*	+ +	−	−	−
pJR37	+ + + +	−	++	+
pJR37-*orf2*	+ +	−	−	+
pJR56	+ + + +	+ +	+ +	+
pJR56-*orf2*	+ + + +	+ +	+ +	+
pJR72	+ + + +	+ +	−	−
pJR76	+ + + +	+	−	+
pJR76-*orf2*	+ + + +	+ +	−	+
pJR125	+ + + +	+ +	−	+
pJR157	+ + + +	+ + +	−	+

*Cultures OD_600_ were adjusted to 1, 10 μL drop of serial dilutions were sequentially spotted on LB-Ap agar plates plus 125 mM sodium perchlorate (NaClO_4_), 0.2 μM 4-nitroquinoline-N-oxide (4-NQO) or 1 mM hydrogen peroxide (H_2_O_2_), or exposed to UV-B radiation at 2 W m^–2^ for 20 s. -, no resistant; +, 1 to 10-fold resistant; ++, 10 to 10^2^-fold resistant; +++, 10^2^ to 10^3^-fold resistant; ++++, more than 10^3^-fold resistant.*

**FIGURE 4:**
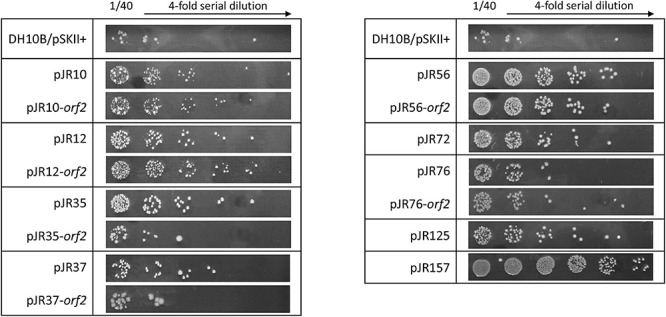
UV-B resistance test by drop assays of the individual predicted ORFs involved in perchlorate resistance and their complete clones. Cultures were grown overnight at 37°C, drops of 4-fold serial dilutions were plated on LB-Ap and plates were incubated for 24 h after exposure to 20 s of UV-B (312 nm) radiation at 2 W m^–2^.

It is widely known that UV radiation produces not only different types of DNA damage, but also oxidative stress via reactive oxidative species (ROS) production (Pattison and Davies, 2006). Therefore, it was tested by drop assay the resistance to UV radiation of *E. coli* DH10B strains carrying the recombinant plasmids with the complete environmental DNA fragments and the individual predicted ORFs conferring perchlorate resistance. For that purpose, cultures of these DH10B strains were plated onto LB-Ap agar medium and exposed to UV-B radiation (312 nm) at 2 W m^–2^ for 20 s (Figure 4). All perchlorate-resistant clones with the complete DNA fragments, except pJR37, showed significant resistance to UV irradiation, with a 9 to 470-fold increase in the survival percentage (between 0.06 and 4%) (Supplementary Figure 2). The same phenomenon was observed for clones harboring the individual ORFs. All of them, except pJR35-*orf2* and pJR37-*orf2*, showed UV-resistance with a survival percentage from 0.11 to 4%, which corresponds to a 13 to 470-fold increase (Supplementary Figure 2). These results may support the biological function of pJR56-*orf2* and pJR157-*orf1*, which encoded for a HU DNA-binding protein and ClpP protease, respectively. HU could protect DNA and ClpP might degrade dysfunctional and misfolded proteins produced by UV radiation.

**FIGURE 5:**
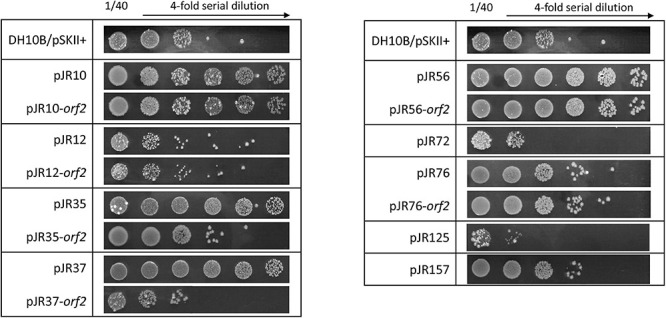
4-NQO (4-nitroquinoline-N-oxide) resistance test by drop assays of the individual predicted ORFs involved in perchlorate resistance and their complete clones. Cultures were grown overnight at 37°C, drops of 4-fold serial dilutions were plated on LB-Ap supplemented with 0.2 μM 4-NQO and plates were incubated for 48 h at 37°C.

Furthermore, we evaluated whether perchlorate-resistant clones showed resistance to the chemical compound 4-NQO, which induces oxidative stress and is supposed to mimic the effect of UV radiation on the DNA (Williams et al., 2010; Han et al., 2017). For that, the same drop assay test was performed as describe above, but in this case DH10B strains harboring perchlorate-resistant DNA fragments or single ORFs were cultured with 0.2 μM 4-NQO (Figure 5). Only pJR10, pJR35, pJR37 and pJR56 clones of the nine DNA fragments involved in perchlorate resistance were resistant to 4-NQO, which showed an increase from 45 to 99-fold in the survival percentage (between 43 and 97%) (Supplementary Figure 3). Furthermore, pJR10-*orf2* and pJR56-*orf2* may induce 4-NQO resistance when cloned in *E. coli* DH10B, showing a survival percentage of 64 and 41%, and a resistance increase of 66 and 42-fold, respectively. These results, together with the data from UV resistance drop assay, suggest that pJR10-*orf2* and pJR56-*orf2* may induce UV-resistance when cloned into DH10B cells by protection or repair of DNA damage, whereas the other UV-resistant ORFs may protect from other types of damage caused by UV radiation, such as pJR157-*orf1*.

**FIGURE 6:**
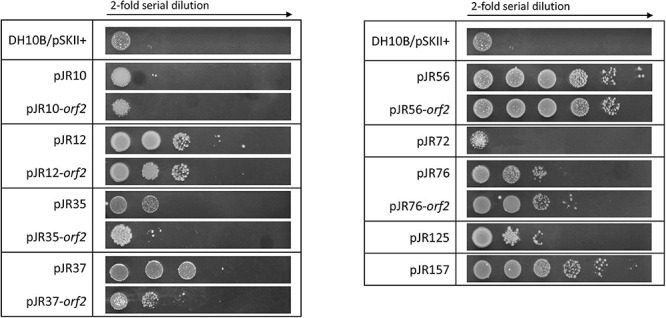
Hydrogen peroxide resistance test by drop assays of the individual predicted ORFs involved in perchlorate resistance and their complete clones. Cultures were grown overnight at 37°C, drops of 2-fold serial dilutions were plated on LB-Ap supplemented with 1 mM H_2_O_2_ and plates were incubated for 24 h at 37°C.

Finally, the effect of hydrogen peroxide (H_2_O_2_), a compound that is known to produce ROS, was also assessed. For this purpose, another drop assay test was performed as before, although perchlorate resistant DH10B strains were exposed to 1 mM H_2_O_2_ (Figure 6). pJR12 and pJR12-*orf2*, pJR37 and pJR37-*orf2*, pJR56 and pJR56-*orf2*, pJR76 and pJR76-*orf2*, pJR125, and pJR157 exhibited significant resistance to 1 mM H_2_O_2_, with a survival percentage from 0.0002 to 0.0013%, which corresponds to a 2 to 17-fold increase (Supplementary Figure 4).

In summary, all environmental DNA fragments and their individual predicted ORFs that conferred resistance to perchlorate, provided resistance to at least other stress condition, such as UV irradiation, exposure to 4-NQO or oxidative stress (Table 2). pJR56 and pJR56-*orf2* conferred resistance to all the tested stress conditions. pJR157-*orf1*, pJR10-*orf2*, and pJR12-*orf2* were the genes that induced the maximum levels of resistance to UV radiation, 4-NQO, and H_2_O_2_, respectively.

## Discussion

To our knowledge, little is known about molecular mechanisms for perchlorate adaptation. In this study, we proposed to search for novel perchlorate-resistant genes in microorganisms from sediments of a hypersaline pond in Salar de Maricunga located at Atacama Desert, the region with the larger natural sources of perchlorate on Earth. Functional metagenomics, as one culture independent technique, would allow for the identification of functional genes from cultivated and uncultivated microorganisms. Although this strategy has been previously used to study mechanisms of adaptation to several extreme conditions, such as acidic pH, metals high salinity or UV-radiation (Mirete et al., 2007, 2015; Kapardar et al., 2010; Culligan et al., 2012, 2013; Guazzaroni et al., 2013; Morgante et al., 2014; Lamprecht-Grandío et al., 2020), this is the first study in which functional metagenomics approach is applied to identify novel perchlorate-resistance genes. A total of nine genes that conferred perchlorate resistance were retrieved from the microbial communities of the sediments of Salar de Maricunga. The fact that these genes would belong to unidentified microorganisms together with the low sequence identities of the gene products remark the novelty of the encoded proteins. The high G + C content of this perchlorate-resistance genes may indicate that most of these genes may have been retrieved from halophilic microorganisms (Jones and Baxter, 2016; González-Torres and Gabaldón, 2018). In fact, closest similar proteins of some of the identified gene products belonged to halophilic bacteria, as *H. ochraceum* (pJR12-*orf2* and pJR35-*orf2*) and *R. ekhonensis* (pJR56-*orf2*). In addition, some of the novel perchlorate-resistance genes encoded for hypothetical proteins with unknown function. These proteins may complement a molecular function previously known to be present in *E. coli*. However, they showed very low or no similarity to the proteins of that microorganism, and therefore they may develop a novel function in the context of *E. coli*.

The toxicity of perchlorate could be related to its chaotropic activity, producing protein denaturation, destabilization of lipid bilayers and precipitation of nucleic acids. Furthermore, perchlorate induces oxidative stress derived from the general damage produced by its chaotropic activity, its oxidative properties, and the unspecific generation of oxidative species such as chlorite and hypochlorite (Ingram, 1981; Hallsworth et al., 2003; Zhang and Cremer, 2010). It is known that an increase in salinity, in this case due to the addition of perchlorate salts, may result in oxidative stress induction even if no oxidant is present (Petrovic, 2006). However, the addition of a similar concentration of sodium chloride, a non-oxidative salt, was completely tolerated by *E. coli* DH10B/pSKII+ (Supplementary Figure 1). Results reported here suggested that the genes identified in this study may be related to a wide variety of process such as DNA protection, degradation of misfolded proteins, RNA processing, tRNA modifications or metabolism. In addition, the overexpression of some of these genes showed an increase in resistance toward UV-radiation, 4-NQO and H_2_O_2_, stress conditions that produce DNA lesions and generate oxidative stress. The identification of genes that conferred resistance to several types of stress may be a result of the adaptation of microorganisms not only to perchlorate, but also to other extreme environmental conditions present in Atacama Desert.

Some of the proteins encoded by the identified ORFs were similar to proteins previously reported to be involved in several stress conditions, such as DNA-binding protein HU (pJR56-*orf2*) and ClpP protease (pJR157-*orf1*). *E. coli* HU is a small basic histone-like protein that constitutes the major protein component of the bacterial nucleoid (Rouvière-Yaniv and Gros, 1975; Rouvière-Yaniv, 1978). It can compact DNA by introducing negative supercoiling into relaxed circular DNA molecules in the presence of topoisomerase I (Rouvière-Yaniv et al., 1979). HU protein participates in several biological processes, and indeed, HU-lacking mutations have pleiotropic effects on the cells. This protein plays an important role in DNA replication and repair and HU-mutants are extremely sensitive to gamma irradiation and UV radiation due to its role in homologous recombination (Boubrik and Rouvière-Yaniv, 1995; Li and Waters, 1998). In addition, HU displays high affinity for some altered DNA structures such as DNA junctions, nicks or gaps even under stringent conditions (Pontiggia et al., 1993; Bonnefoy et al., 1994; Castaing et al., 1995; Kamashev et al., 1999; Pinson et al., 1999). When pJR56-*orf2* was cloned into DH10B strain, it was shown an increase in the resistance to perchlorate, UV-radiation, 4-NQO, and H_2_O_2_. Since these types of stress produce DNA damage directly or indirectly, it may be suggested that these results are due to the role of HU in DNA protection or repair. Furthermore, it was described that HU is required for efficient expression of the RNA polymerase sigma S subunit RpoS (Balandina et al., 2001). RpoS induces the expression of most of the genes involved in survival under starvation and stress conditions (Lange and Hengge-Aronis, 1991; Loewen and Hengge-Aronis, 1994). In addition, in previous studies of this laboratory it was shown that an environmental DNA fragment encoding for a protein similar to another HU, conferred resistance to acidic conditions (Guazzaroni et al., 2013). Therefore, HU may trigger a global stress response by inducing RpoS expression that could increase the resistance against a wide variety of stress conditions.

On the other hand, Clp proteases are involved in the degradation of misfolded proteins, the regulation of short-lived proteins and the removal of dysfunctional proteins (Chandu and Nandi, 2004). In *E. coli*, ClpP subunit associates with ClpA or ClpX to form a large complex with substrate-specific protease activity (Gottesman, 1996). Overexpression of pJR157-*orf1* in *E. coli* conferred resistance to perchlorate, UV radiation and H_2_O_2_. ClpP protease may contribute to the degradation of proteins denaturized by the chaotropic activity of perchlorate and oxidative stress. Furthermore, in other studies of this laboratory, it was reported that ClpP protease and other Clp proteins are involved in resistance to UV radiation and other stress conditions in *E. coli* (Guazzaroni et al., 2013; Morgante et al., 2014). It is widely known that UV radiation produces not only DNA damage, but also protein damage via ROS production (Pattison and Davies, 2006). As overexpression the putative ClpP protease encoded by pJR157-*orf1* did not confer resistance to 4-NQO, its role in DNA protection or repair could be excluded.

Other genes found in this study encoded for proteins involved in RNA metabolism: a QueF enzyme (pJR12-*orf2*), a Hfq-domain containing hypothetical protein (pJR10-*orf2*) and a truncated RNase E (pJR35-*orf2*).

The pJR12-*orf2* encoded for a protein very similar to the QueF enzyme from *H. ochraceum*. When cloned into *E. coli* DH10B, pJR12-*orf2* overexpression increased resistance to perchlorate, UV-radiation or H_2_O_2_. QueF enzymes catalyze the NADPH-dependent reduction of the nitrile group of 7-cyano-7-deazaguanine (preQ_0_) to 7-aminomethyl-7-deazaguanine (preQ_1_) in the queuosine (Q) biosynthetic pathway (Van Lanen et al., 2005). Q is a modified nucleoside that is incorporated in the position 34 of certain tRNAs containing the GUN anticodon (those encoding for His, Asn, Tyr, and Asp) (Harada and Nishimura, 1972). The precise physiological role of Q has not been established to date. Q is known to be related to stress tolerance in eukaryotes and bacteria. In mammalian cells, Q promotes the antioxidant defense system by increasing catalase activity, and modulates tolerance to hypoxia (Reisser et al., 1994; Pathak et al., 2007). In addition, *Drosophila melanogaster* embryos were more sensible to cadmium when Q was absent (Siard et al., 1991). In *E. coli*, the absence of Q reduced viability when cells were cultured under conditions unsuitable for growth (Noguchi et al., 1982), and an increase in resistance to several stress conditions was shown (arsenic, UV-radiation, low acidic pH and heat shock) when genes from environmental microorganisms that encode for enzymes that catalyze the last two steps of the Q-tRNA modification pathway were overexpressed (Morgante et al., 2014). Considering these data, it is not surprising that overexpression of pJR12-*orf2* allows for protection against oxidative stress and other types of damage produced by perchlorate, UV-radiation or H_2_O_2_.

There are two subfamilies of QueF, type I that are homodecameric enzymes of unimodular subunits, and type II that are larger and form lower-order quaternary structures of bimodular subunits (Van Lanen et al., 2005). Considering the similarity of the protein encoded by pJR12-*orf2* with *B. subtilis* QueF (type I) but not with the *E. coli* QueF (type II), it can be suggested that this QueF protein could be classified as a type I protein. In addition, it is known that a conserved cysteine of the QueF active site is prone to oxidation, leading to inactivation of the enzyme. However, in type I proteins, this residue is protected from irreversible oxidation by a conserved intramolecular disulfide bond (Mohammad et al., 2017). Therefore, the protein encoded by pJR12-*orf2* and *B. subtilis* QueF could be protected from oxidative stress produced by perchlorate. In fact, when *queF* genes from *E. coli* and *B. subtilis* were overexpressed in *E. coli* DH10B, it was observed that only *B. subtilis queF* gene conferred perchlorate resistance. These data would confirm that pJR12-*orf2* encoded for a type I QueF and that these proteins are protected against oxidation.

Continuing with other proteins related to RNA metabolism, Hfq-domain containing hypothetical protein (pJR10-*orf2*) conferred perchlorate and UV-radiation resistance. Hfq (pJR10-*orf2*) is one of the major post-transcriptional regulators in bacteria. It acts as an RNA chaperone, remodeling RNA secondary structures and promoting RNA–RNA interactions (Dos Santos et al., 2019). Disruption of Hfq is known to reduce bacterial growth and alter mutagenesis, leading to sensitivity to several stresses, such as UV radiation, oxidants and osmotic shock (Tsui et al., 1994). In addition, Hfq protein is required to induce the translation of RpoS (Brown and Elliott, 1996). Therefore, these data may support that the overexpression of pJR10-*orf2* protects against perchlorate and UV radiation.

The main role of Hfq is to promote the interaction between small non-coding RNAs (sRNAs) and their mRNAs targets. Most of these sRNA/mRNA interactions lead to the inhibition of translation, usually by triggering mRNA decay (Massé et al., 2003; Dos Santos et al., 2019). This process is mediated by Hfq and RNase E, which has also been identified in this study. The pJR35-*orf2* produces a truncated RNase E containing the amino-terminal part of the protein where ribonuclease activity resides. In one model, it is proposed that RNase E associates with the chaperone Hfq, and then, Hfq binds a sRNA and subsequently directs the RNase E–Hfq–sRNA complex to target mRNAs by specific sRNA–mRNA base-pairing (Aiba, 2007). Bound RNase E cleaves targeted mRNA and triggers mRNA decay. However, the truncated RNAse E could not associate with Hfq since it lacks the carboxy-terminal region involved in the interaction of the two proteins (Mackie, 2013). In an alternative model, initial sRNA–mRNA interaction is independent of RNase E. Hfq would promote sRNA–mRNA base-pairing, rendering targeted mRNA more susceptible to RNase E cleavage (Bandyra et al., 2012). This model would be compatible with the truncated protein encoded by pJR35-*orf2*. It should be noted that pJR35-*orf2* conferred lower perchlorate resistance than pJR35 harboring the complete environmental DNA fragment. The pJR35-*orf1* encoded for a C-terminal truncated pseudouridine synthase RluA from *H. ochraceum*. This enzyme modifies uridine into pseudouridine at certain positions of the 23S rRNA and tRNAs (Raychaudhuri et al., 1999). It was suggested that these modifications play a role in the stability of RNA and ribosome biogenesis (Cabello-Villegas and Nikonowicz, 2005; O’Connor et al., 2017). Furthermore, RNase E participates in rRNA and tRNA maturing processes (Mackie, 2013), and therefore it could increase the levels of these RNA molecules. Thus, the RluA pseudouridine synthase encoded by pJR35-*orf1* may help to stabilize mature rRNA and tRNA produced by RNase E. An increase not only in the levels but also in the stability of rRNA and tRNA could enhance ribosome assembly and protein biosynthesis, which may help to resist the nucleic acid denaturalization produced by the chaotropic effects of perchlorate. Considering that overexpression of both pJR10-*orf2* and pJR35-*orf2* increased resistance to perchlorate, it may be suggested that translation regulation by sRNAs, Hfq, and RNase E may be a key process for enhancing a cellular stress response.

Another gene identified in this study was pJR37-*orf2*, which encodes for a truncated hypothetical protein containing an alanine dehydrogenase domain. Alanine dehydrogenase (Ald) catalyzes the NAD(H)-dependent reversible reductive amination of pyruvate into L-alanine (Dave and Kadeppagari, 2019). The overexpression of pJR37-*orf2* increased the resistance levels of *E. coli* DH10B to perchlorate and H_2_O_2_. Aerobic respiration is known to be the major source of ROS and the cellular respiration machinery is reduced by oxidative stress (Kang et al., 2013; Larosa and Remacle, 2018). Under respiratory-inhibiting conditions, *ald* is induced to maintain the redox balance of the NAD^+^/NADH pool by reductive amination of pyruvate (Jeong and Oh, 2019). In *E. coli*, this function is mainly performed by lactate dehydrogenase, but *ald* overexpression was able to complement a lactate dehydrogenase mutant (Giffin et al., 2016). Therefore, the oxidative stress produced by perchlorate or H_2_O_2_ may decrease aerobic respiration to reduce direct ROS production, and the overexpression of pJR37-*orf2* might help to maintain the redox balance in this situation. Furthermore, we observed that pJR37-*orf2* conferred lower perchlorate resistance than pJR37. This data suggests that pJR37-*orf1* could be necessary for an optimal transcription and RNA stabilization of pJR37-*orf2* or for maintaining the function of the protein encoded by pJR37-*orf2* by an unknown mechanism.

The truncated protein encoded by pJR72-*orf1* was similar to an inosine 5-monophosphate dehydrogenase (IMPDH), and conferred resistance to perchlorate and UV-radiation when cloned into DH10B strain. IMPDH catalyzes the NAD^+^-dependent oxidation of IMP to XMP, the pivotal step in the *de novo* biosynthesis of guanine nucleotides. Furthermore, there is evidence that IMPDH from *E. coli* and some eukaryotes can bind single-stranded nucleic acids through their CBS domain (McLean et al., 2004). Under oxidative stress, it accumulates in the nucleus and represses cell proliferation genes in *D. melanogaster* by binding CT-rich single-stranded unwound DNA sequences in those genes (Kozhevnikova et al., 2012). The IMPDH encoded by pJR72-*orf1* was similar to its *E. coli* homolog (46%) and also contained a CBS domain. Thus, we suggest that it could play a role in regulating the expression of certain genes for protection against some oxidative stress conditions.

Finally, two more genes were identified encoding for hypothetical proteins that conferred resistance to perchlorate and to H_2_O_2_. The protein encoded by pJR125-*orf1* displayed low similarity to the closest BLASTP hit, a hypothetical protein from a microorganism of the *Deltaproteobacteria* class. Neither a conserved domain nor transmembrane helices nor signal peptides could be predicted, no putative function to this novel protein could be provided. On the other hand, pJR76-*orf2* encoded for a protein similar (52% identity) to another hypothetical protein from a microorganism of the *Gammaproteobacteria* class. However, this protein contained a domain belonging to the MlaC family. MlaC is a component of the Mla pathway, an ABC transporter system that functions to maintain the asymmetry of the outer membrane in Gram-negative bacteria (Ekiert et al., 2017). The outer membrane of *E. coli* is an asymmetric bilayer with lipopolysaccharides (LPSs) localized in the outer leaflet and phospholipids (PLs) in the inner leaflet (Nikaido, 2005). This lipid asymmetry is essential for the barrier function of the OM and disruption of the asymmetric composition reduces the effectiveness of the barrier and cells become more sensitive to various stressful conditions (Malinverni and Silhavy, 2009). It is known that in MlaC is involved in toluene tolerance in *Pseudomonas putida* and is up-regulated under high salinity conditions in the extreme acidophile *Acidihalobacter prosperus* (Kim et al., 1998; Dopson et al., 2017). Perchlorate and H_2_O_2_ induce oxidative stress, which may lead to lipid damage and impaired membrane integrity. Thus, it can be proposed that the protein encoded by pJR76-*orf2* induced perchlorate resistance due to its function as a MlaC protein helping to maintain lipid asymmetry and integrity. However, when the *E. coli yrbC* encoding MlaC was overexpressed in DH10B strain, no perchlorate resistance was observed. One possible explanation is that the protein encoded by pJR76-*orf2* was not similar (less than 30% identity) to the *E. coli* MlaC. Furthermore, although they shared a conserved domain, *E. coli* MlaC is a globular periplasmic protein, whereas the hypothetical protein contained one predicted transmembrane domain. Thus, the protein encoded by pJR76-*orf2* may have another function related to phospholipid traffic.

The discovery of nine novel perchlorate-resistance genes illustrates that this methodology has been applied successfully. This work has revealed a wide variety of processes that could be related to perchlorate resistance, such as DNA protection and repair, RNA processing, tRNA modification, protein degradation and metabolism. Therefore, tolerance to perchlorate may involve a global cellular response. Furthermore, some of the proteins encoded by novel perchlorate-resistance genes are hypothetical proteins that may show an unknown function not performed by any of the *E. coli* proteins. Nevertheless, further characterization of the identified genes will improve understanding of the molecular mechanisms and metabolic pathways involved in perchlorate resistance, and it will also open the way to design relevant biotechnological applications for Mars exploration. As mentioned before, liquid brines and regolith on Mars are known to contain high levels of perchlorate, threatening human health, plant growth and the development of Bioregenerative Life Support Systems (BLSS) in future explorations and the colonization of this planet. Due to the long duration of these trips and their high cost, it is mandatory to develop BLSS based on *in situ* resources utilization (ISRU) for the production of oxygen and food by plants and phototrophic microorganisms and the recycling of waste material, as well as other biotechnological application to obtain construction material and for biomining. The use of microorganisms in BLSS has been proposed, because they would need relatively fewer requirements and it is known that several species present molecular mechanisms to perform those processes (Sridhar et al., 2000; Rothschild, 2016; Averesch and Rothschild, 2019; Billi et al., 2020). However, the presence of high perchlorate concentrations in the Martian soil, along with high UV-radiation and desiccation, suppose a limiting factor for BLSS. Thus, these novel perchlorate resistance genes may be used in synthetic biology to create perchlorate resistance strains of interest in BLSS, and could enable the use of perchlorate-rich Martian water and soil without the need for perchlorate remediation method, which are technologically demanding and highly expensive (Coates and Achenbach, 2004; Montague et al., 2012).

## Data Availability Statement

The datasets presented in this study can be found in onlinerepositories. The names of the repository/repositories and accession number(s) can be found below: https://www.ncbi.nlm.nih.gov/nuccore/mw924778; https://www.ncbi.nlm.nih.gov/nuccore/mw924779; https://www.ncbi.nlm.nih.gov/nuccore/mw924780; https://www.ncbi.nlm.nih.gov/nuccore/mw924781; https://www.ncbi.nlm.nih.gov/nuccore/mw924782; https://www.ncbi.nlm.nih.gov/nuccore/mw924783; https://www.ncbi.nlm.nihgov/nuccore/mw924784; https://www.ncbi.nlm.nih.gov/nuccore/mw924785; and https://www.ncbi.nlm.nih.gov/nuccore/mw924786.

## Author Contributions

JD-R and JG-P designed the experiments and wrote the manuscript. JD-R constructed the metagenomic library, performed the screening, the bioinformatic analysis, and the different tests of the resistant clones. GR-V, FT-R, LC, IV, and BG collaborated in the collection of the environmental samples and in the revision of the manuscript. All authors contributed to the article and approved the submitted version.

## Conflict of Interest

The authors declare that the research was conducted in the absence of any commercial or financial relationships that could be construed as a potential conflict of interest.

## Publisher’s Note

All claims expressed in this article are solely those of the authors and do not necessarily represent those of their affiliated organizations, or those of the publisher, the editors and the reviewers. Any product that may be evaluated in this article, or claim that may be made by its manufacturer, is not guaranteed or endorsed by the publisher.

## Footnotes

1 https://www.ncbi.nlm.nih.gov/orffinder/

2 https://pfam.xfam.org/

3 http://smart.embl-heidelberg.de/

4 https://www.ncbi.nlm.nih.gov/Structure/cdd/wrpsb.cgi

5 https://embnet.vital-it.ch/software/TMPRED_form.html

## Supplementary Material

Supplementary Figure 1 Drop assay in the presence of perchlorate and sodium chloride of *E. coli* DH10B/pSKII+. Cultures were grown overnight at 37°C, drops of 10-fold serial dilutions were plated on LB-Ap containing 125 mM of sodium perchlorate or sodium chloride and plates were incubated for 48 h at 37°C.

Supplementary Figure 2 Survival percentage after UV-B exposure of the individual predicted ORFs involved in perchlorate-resistance and their complete clones. Cultures were grown overnight at 37°C, drops of 4-fold serial dilutions were plated on LB-Ap agar and plates were incubated for 24 h after exposure to 20 s of UV-B (312 nm) radiation at 2 W m^–2^. Survival percentage was calculated as the number of CFU mL^–1^ remaining after the treatment divided by the initial CFU mL^–1^. *E. coli* DH10B bearing empty pSKII+ (pBlueScript II SK+) vector was used as a negative control. One-way ANOVA followed by Dunnet *post hoc* analysis revealed the displayed significant differences against the negative control (^∗^*p* < 0.05; ^∗∗^*p* < 0.01; ^∗∗∗^*p* < 0.001). Data represent the mean ± S.D (*n* ≥ 3).

Supplementary Figure 3 Survival percentage in the presence of 4-NQO of the individual predicted ORFs involved in perchlorate-resistance and their complete clones. Cultures were grown overnight at 37°C, drops of 4-fold serial dilutions were plated on LB-Ap agar supplemented with 0.2 μM 4-NQO and plates were incubated for 48 h at 37°C. Survival percentage was calculated as the number of CFU mL^–1^ remaining after the treatment divided by the initial CFU mL^–1^. *E. coli* DH10B bearing empty pSKII+ (pBlueScript II SK+) vector was used as a negative control. One-way ANOVA followed by Dunnet *post hoc* analysis revealed the displayed significant differences against the negative control (^∗^*p* < 0.05; ^∗∗^*p* < 0.01; ^∗∗∗^*p* < 0.001). Data represent the mean ± S.D (*n* ≥ 3).

Supplementary Figure 4 Survival percentage in the presence of hydrogen peroxide of the individual predicted ORFs involved in perchlorate-resistance and their complete clones. Cultures were grown overnight at 37°C, drops of 2-fold serial dilutions were plated on LB-Ap agar supplemented with 1 mM H_2_O_2_ and plates were incubated for 24 h at 37°C. Survival percentage was calculated as the number of CFU mL^–1^ remaining after the treatment divided by the initial CFU mL^–1^. *E. coli* DH10B bearing empty pSKII+ (pBlueScript II SK+) vector was used as a negative control. One-way ANOVA followed by Dunnet *post hoc* analysis revealed the displayed significant differences against the negative control (^∗^*p* < 0.05; ***p* < 0.01; ^∗∗∗^*p* < 0.001). Data represent the mean ± S.D (*n* ≥ 3).
