# Supplementary figures and images for: Mining for Perchlorate Resistance Genes in Microorganisms From Sediments of a Hypersaline Pond in Atacama Desert, Chile
Source: Front Microbiol. 2021 Jul 23;12:723874. doi: 10.3389/fmicb.2021.723874 (PMC8343002; doi:10.3389/fmicb.2021.723874)

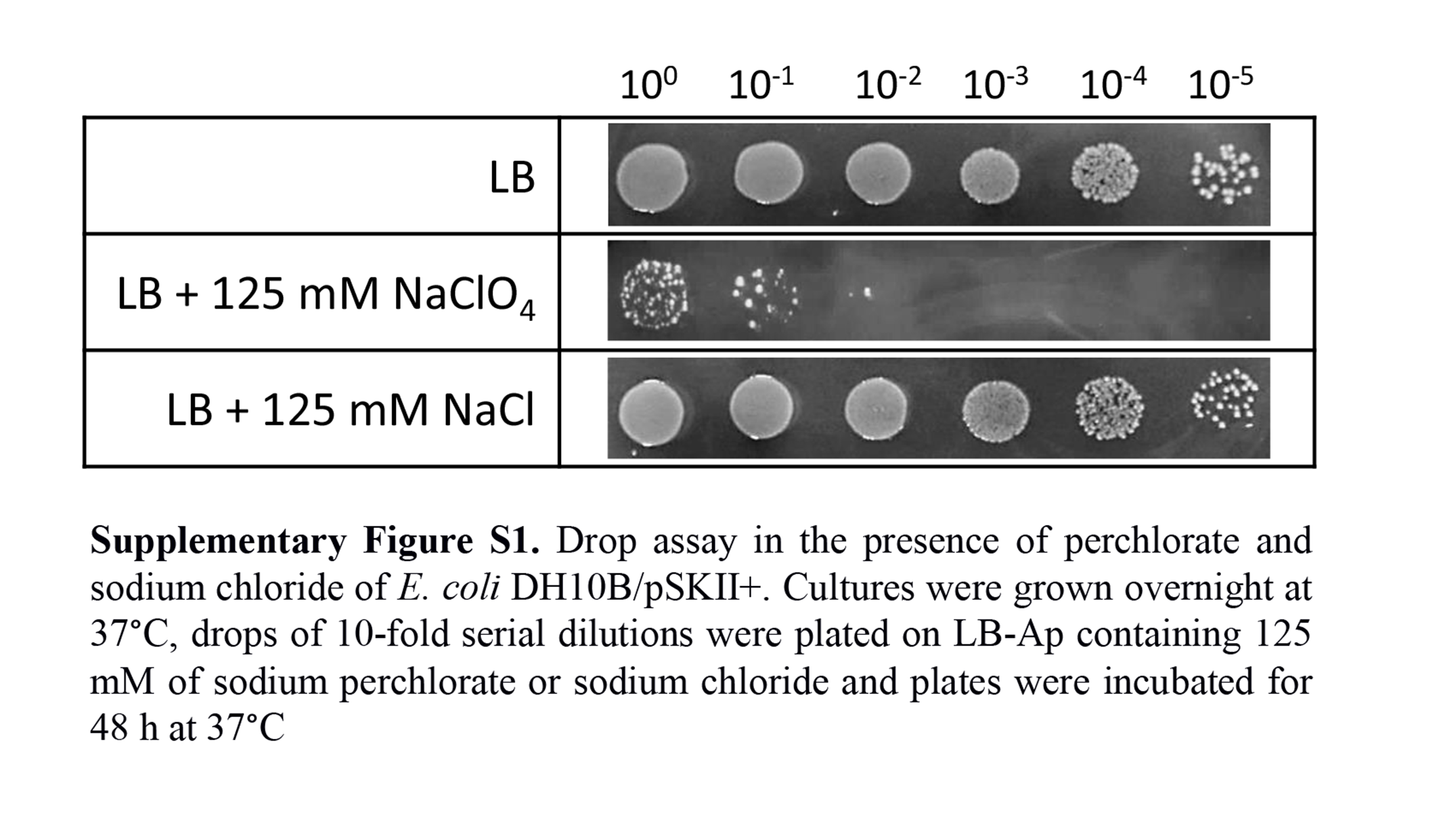

Supplement: Supplementary Figure 1 — Drop assay in the presence of perchlorate and sodium chloride of E. coli DH10B/pSKII+. Cultures were grown overnight at 37°C, drops of 10-fold serial dilutions were plated on LB-Ap containing 125 mM of sodium perchlorate or sodium chloride and plates were incubated for 48 h at 37°C. [file Image_1.TIF]

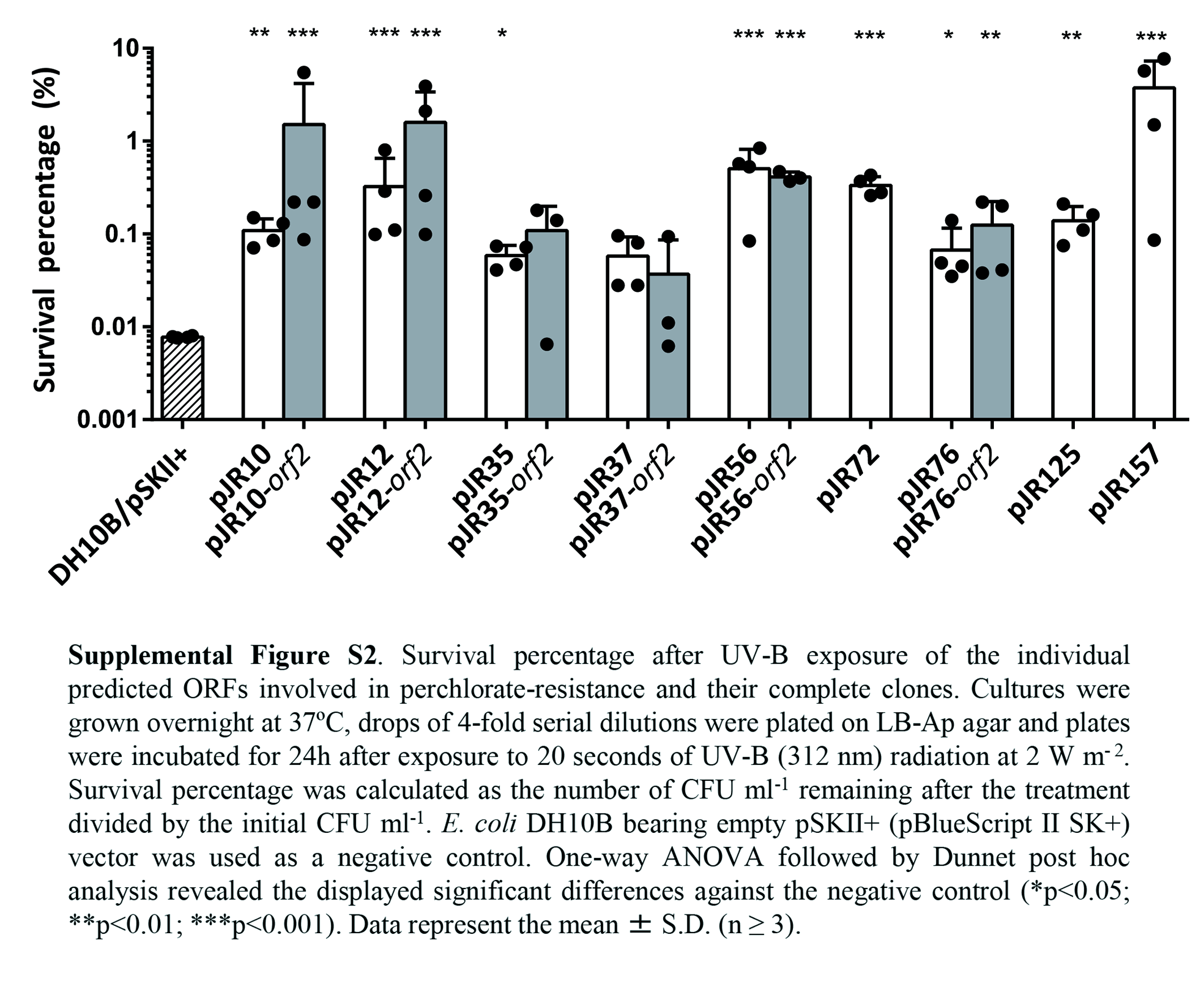

Supplement: Supplementary Figure 2 — Survival percentage after UV-B exposure of the individual predicted ORFs involved in perchlorate-resistance and their complete clones. Cultures were grown overnight at 37°C, drops of 4-fold serial dilutions were plated on LB-Ap agar and plates were incubated for 24 h after exposure to 20 s of UV-B (312 nm) radiation at 2 W m–2. Survival percentage was calculated as the number of CFU mL–1 remaining after the treatment divided by the initial CFU mL–1. E. coli DH10B bearing empty pSKII+ (pBlueScript II SK+) vector was used as a negative control. One-way ANOVA followed by Dunnet post hoc analysis revealed the displayed significant differences against the negative control (∗p < 0.05; ∗∗p < 0.01; ∗∗∗p < 0.001). Data represent the mean ± S.D (n ≥ 3). [file Image_2.TIF]

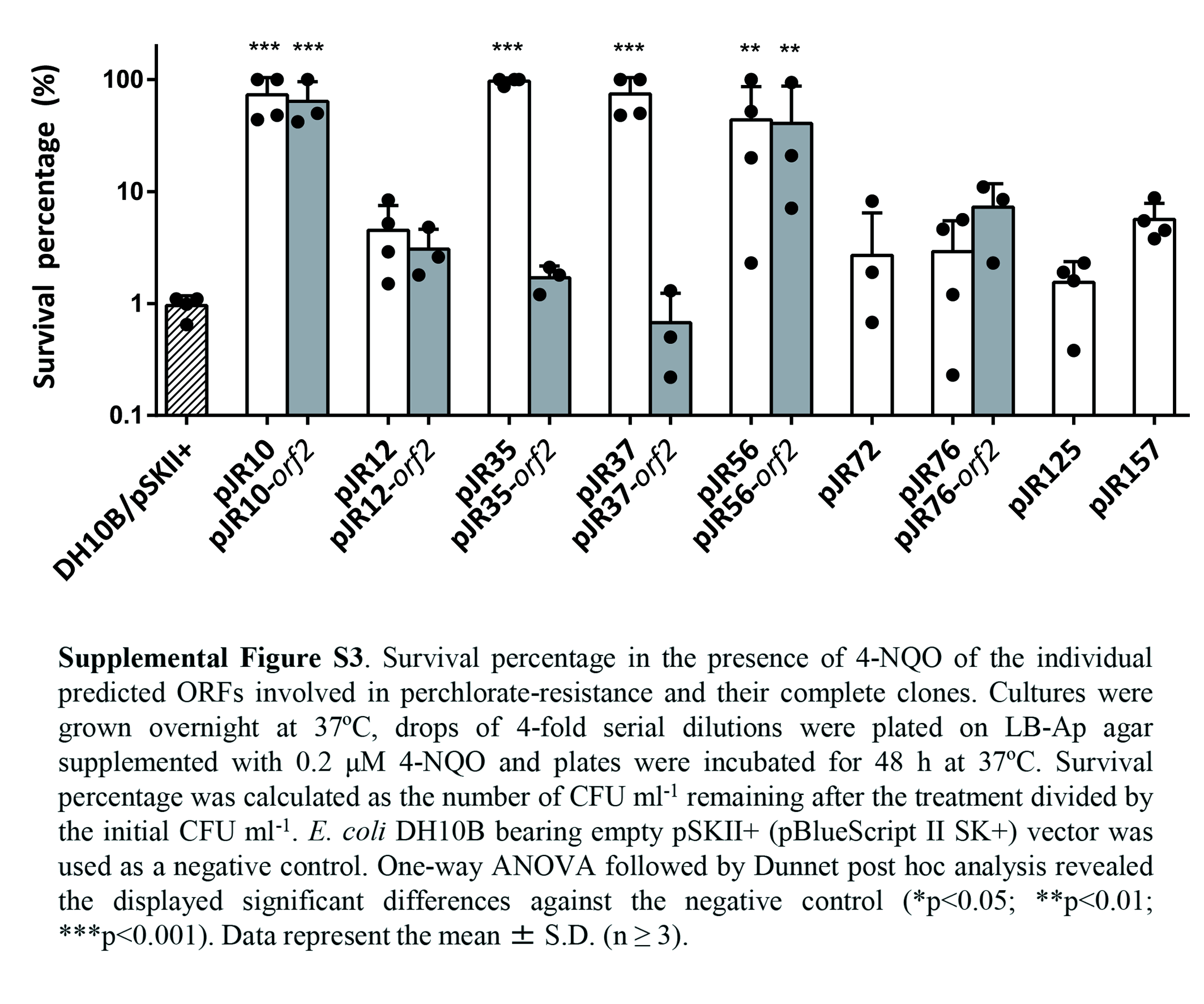

Supplement: Supplementary Figure 3 — Survival percentage in the presence of 4-NQO of the individual predicted ORFs involved in perchlorate-resistance and their complete clones. Cultures were grown overnight at 37°C, drops of 4-fold serial dilutions were plated on LB-Ap agar supplemented with 0.2 μM 4-NQO and plates were incubated for 48 h at 37°C. Survival percentage was calculated as the number of CFU mL–1 remaining after the treatment divided by the initial CFU mL–1. E. coli DH10B bearing empty pSKII+ (pBlueScript II SK+) vector was used as a negative control. One-way ANOVA followed by Dunnet post hoc analysis revealed the displayed significant differences against the negative control (∗p < 0.05; ∗∗p < 0.01; ∗∗∗p < 0.001). Data represent the mean ± S.D (n ≥ 3). [file Image_3.TIF]

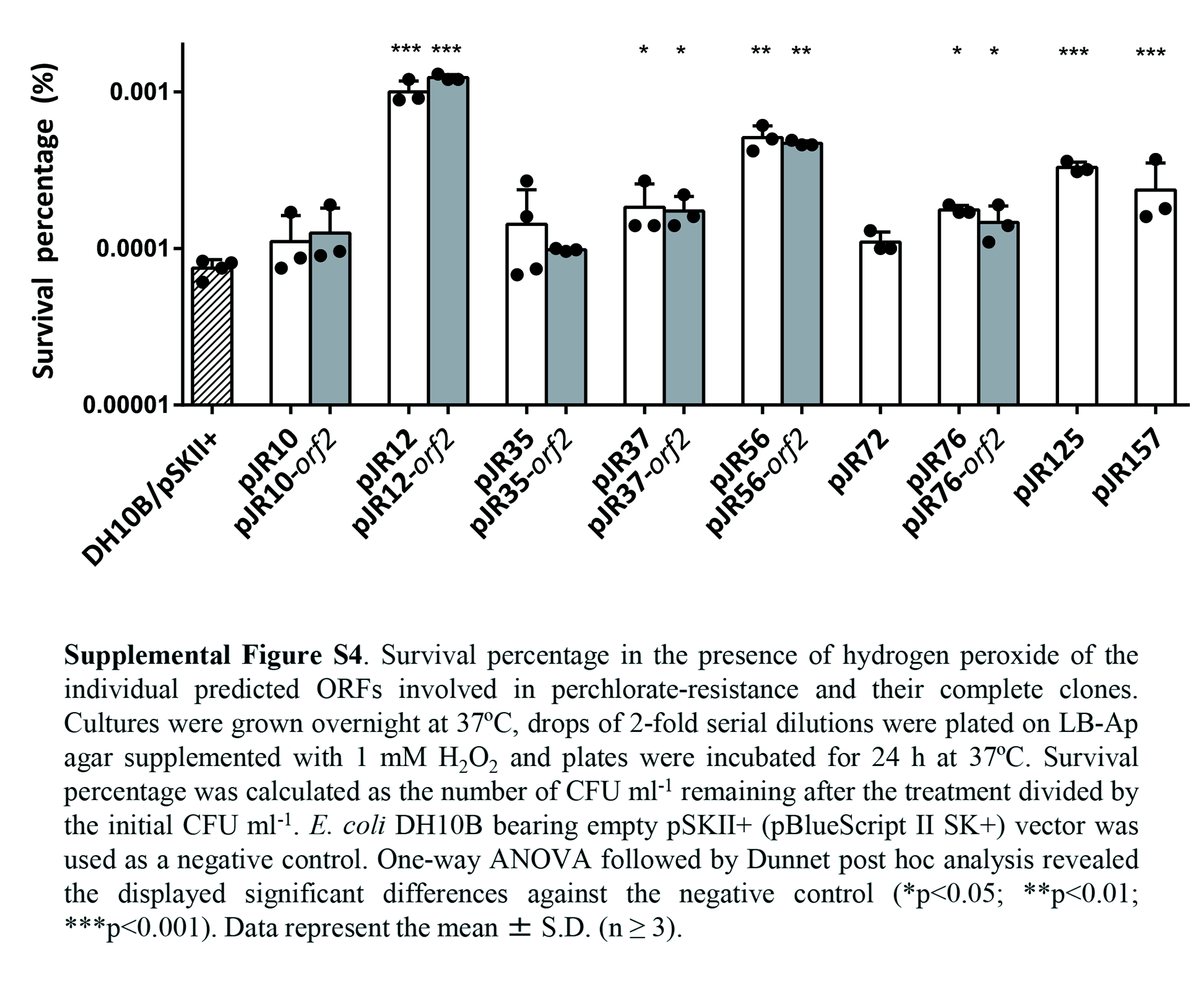

Supplement: Supplementary Figure 4 — Survival percentage in the presence of hydrogen peroxide of the individual predicted ORFs involved in perchlorate-resistance and their complete clones. Cultures were grown overnight at 37°C, drops of 2-fold serial dilutions were plated on LB-Ap agar supplemented with 1 mM H2O2 and plates were incubated for 24 h at 37°C. Survival percentage was calculated as the number of CFU mL–1 remaining after the treatment divided by the initial CFU mL–1. E. coli DH10B bearing empty pSKII+ (pBlueScript II SK+) vector was used as a negative control. One-way ANOVA followed by Dunnet post hoc analysis revealed the displayed significant differences against the negative control (∗p < 0.05; **p < 0.01; ∗∗∗p < 0.001). Data represent the mean ± S.D (n ≥ 3). [file Image_4.TIF]

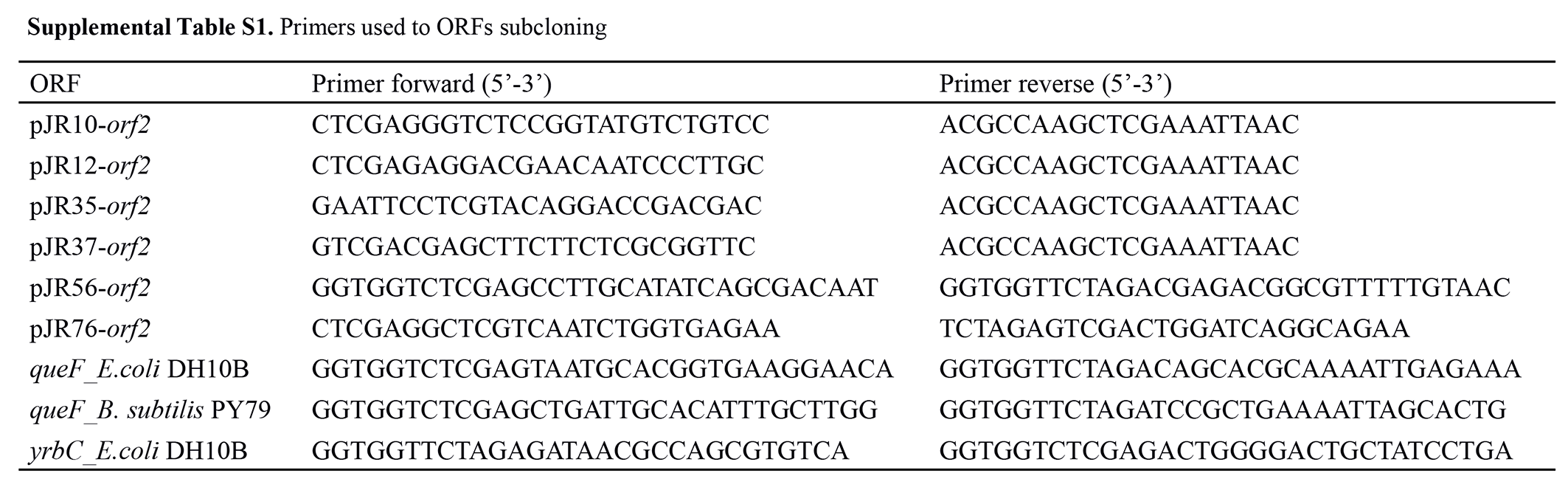

Supplement: Supplementary file 5 [file Image_5.TIF]
